# Draft Genome Sequences of Human Pathogenic Fungus *Geomyces pannorum Sensu Lato* and Bat White Nose Syndrome Pathogen *Geomyces *(*Pseudogymnoascus*) *destructans*

**DOI:** 10.1128/genomeA.01045-13

**Published:** 2013-12-19

**Authors:** Marcus C. Chibucos, Jonathan Crabtree, Sushma Nagaraj, Sudha Chaturvedi, Vishnu Chaturvedi

**Affiliations:** Institute for Genome Sciences, University of Maryland School of Medicine, Baltimore, Maryland, USAa; Department of Microbiology and Immunology, University of Maryland School of Medicine, Baltimore, Maryland, USAb; Mycology Laboratory, Wadsworth Center, New York State Department of Health, Albany, New York, USAc; Department of Biomedical Sciences, School of Public Health, University at Albany, Albany, New York, USAd

## Abstract

We report the draft genome sequences of *Geomyces pannorum sensu lato* and *Geomyces (Pseudogymnoascus) destructans*. *G. pannorum* has a larger proteome than *G. destructans*, containing more proteins with ascribed enzymatic functions. This dichotomy in the genomes of related psychrophilic fungi is a valuable target for defining their distinct saprobic and pathogenic attributes.

## GENOME ANNOUNCEMENT

*Geomyces pannorum* is a soil-dwelling fungus common in colder parts of the world (1–3). *G. pannorum* is rarely implicated in the human disease geomycosis, which manifests as skin and nail infections (4–6). *Geomyces destructans* is the etiologic agent of bat geomycosis or white nose syndrome (WNS) (7, 8). *G. destructans* is restricted to caves and mines in the United States and Europe (9, 10). *G. pannorum sensu lato* represents a species complex, while *G. destructans* was recently reclassified as *Pseudogymnoascus destructans* (2, 9). As taxonomy is in a state of flux, *G. destructans* and *G. pannorum* will be used as the organism names throughout this work. Both fungi are adapted to a psychrophilic range (4° to 15°C) and express enzymes implicated in fungal virulence (1, 3, 8, 11). The study of the biology and pathogenicity of psychrophilic fungi is in its infancy due to a lack of experimental tools. This is also true for other eukaryotes inhabiting colder parts of the earth.

Genomic DNA from *G. pannorum* M1372 and *G. destructans* M1379 was obtained by phenol-chloroform extraction of pulverized fungal mycelia. The Illumina HiSeq 2000 was used for 100-base paired-end sequencing. The assemblies were generated using four programs, subsampling 89 million reads for *G. pannorum* and 88 million reads for *G. destructans*. Optimal assemblies representing 100× coverage for *G. pannorum* and 150× coverage for *G. destructans* were generated with MaSuRCA version 1.9.2 (12). The *G. pannorum* assembly is 29.47 Mb, with a G+C content of 50.5%, and comprises 856 scaffolds ranging in length from 300 bases to 839 kb (average, 34.4 kb; median, 7.9 kb; N_50_, 105 kb). *G. destructans* is 30.49 Mb, with a G+C content of 49.8%, and was assembled into 5,008 scaffolds of length 108 bases to 234 kb (average, 6.1 kb; median, 1.8 kb; N_50_, 19.2 kb). Fifty-two percent of the *G. destructans* reference DNA shares similarity with *G. pannorum* query DNA, with 45% of it being identical. A custom *ab initio* gene prediction pipeline generated 9,689 *G. pannorum* proteins and 7,967 *G. destructans* proteins. The difference in the coding density, which is 48% for *G. pannorum* and 37% for *G. destructans*, is attributable to the smaller number of proteins and numerous repeats (25.8% of the genome) in *G. destructans* than in *G. pannorum* (5.40% repeats). CEGMA predicted 96.7% of the 458 conserved genes in *G. destructans* and 98.3% in *G. pannorum* (13). Functional annotation with BLAST against UniProt (14) and HMM searches against TIGRfams/Pfams (15, 16) yielded Gene Ontology annotations (17) for 62.3% of the *G. destructans* proteins and 63.1% of the *G. pannorum* proteins. EC assignments were given to 2,052 *G. destructans* proteins (25.8%) and 2,734 *G. pannorum* proteins (28.2%). Thus, *G. pannorum* contains more proteins than *G. destructans*, including more with ascribed enzymatic functions. This dichotomy in the genomes of related psychrophilic fungi is a valuable target for defining their distinct saprobic and pathogenic attributes.

### Nucleotide sequence accession numbers.

The *G. (Pseudogymnoascus) destructans* whole-genome shotgun project has been deposited at DDBJ/EMBL/GenBank under the accession no. AYKP00000000. The version described in this paper is AYKP01000000. The *G. pannorum* whole-genome shotgun project has been deposited at DDBJ/EMBL/GenBank under the accession no. AYKR00000000. The version described in this paper is AYKR01000000.
